# Length doesn’t play a role – Sexual activity in men after short stem Total hip Arthroplasty

**DOI:** 10.1186/s12891-022-05660-8

**Published:** 2022-07-22

**Authors:** Luis Navas, Matthias Hauschild, Wolfgang Miehlke, Sebastian Schmidt, Marcus Streit, Stefan Kinkel, Alexander Zimmerer

**Affiliations:** 1 ARCUS Sportklinik, Rastatterstraße 17-19, 72175 Pforzheim, Germany; 2 Orthopädische Klinik Paulinenhilfe, Diakonieklinikum Stuttgart, Rosenbergstraße 38, 70176 Stuttgart, Germany; 3 Department of Orthopedics and Orthopedic Surgery, University Medicine Greifswald, Ferdinand-Sauerbruch-Straße, 17475 Greifswald, Germany

**Keywords:** Hip Arthroplasty, Sexual activity, Sexual positions, Patient-reported outcomes

## Abstract

**Background:**

Chronic hip pain due to osteoarthritis or hip dysplasia has been shown to negatively affect many daily life aspects. One aspect, however, which persists underestimated is sexual health. The number of total hip arthroplasties (THA) are increasing, especially in young patients who have high functional expectations, not only to pain relief, but to an increase in hip mobility and quality of life as well as sexual activity.

**Aim:**

(1) to report the demographic factors, (2) the sexual activity before and after THA, as well as the concerns related to sexual activity after THA and (3) the patient-reported outcome measurements (PROMs) in sexually active male patients.

**Methods:**

We evaluated the results of patients between 18 and 65 years of age following primary cementless short femoral stem THA using a direct anterior approach (DAA) at a midterm follow-up of 4 years. A web-based questionnaire (via SurveyMonkey) was chosen to assess frequency, positions, complaints, fears, dealing with the questions and PROMs. Our patients sexual activity was via The Sexual Health Inventory for Men (SHIM) validated.

**Results:**

Patients resumed their sexual activities after 6 weeks. The two main causes of difficulty in sexual activity before surgery were pain and limitation of the range of motion of the hip joint. Patients experienced less pain and an improvement in hip range of motion after THA. 89% of patients expressed a desire for more detailed and specific information on the subject. The patients foremost concern about muscle weakness, surgical scar or fear of dislocation. After 4 years follow-up our patients presented a significant improvement of the modified Harris Hip Score (mHHS) from 34.1 preoperative to 92.6 after THA.

**Conclusion:**

THA improves the quality in sexual life, in relation to less pain and improvement in the range of motion, but not in the frequency of sexual activity. Men’s sexual positions required less mobility and could therefore be considered safer.

## Introduction

Chronic hip pain due to osteoarthritis or hip dysplasia has been shown to negatively affect many daily life aspects. One aspect which persists underestimated is the sexual health of the patients [1]. Nowadays, sexual activity remains a difficult and embarrassing topic for both patient and physician [2]. Therefore, many surgeons do not address the topic during the consultation, resulting in the patient’s symptoms and concerns not being adequately addressed. In some cases of hip pain, difficulties with sexual activity may be one of the main reasons for consultation [3, 4]. The World Health Organization (WHO) defines sexual health as “a state of physical, emotional, mental and social well-being in relation to sexuality” [5], thereby establishing a strong relationship between general well-being and the quality of sexual life. With regard to the above, many studies have shown lower levels of sexual fulfillment when osteoarthrosis of the hip is present [6–8]. On this basis, indications for total hip arthroplasty (THA) are increasing, especially in young patients who have high functional expectations, not only to pain relief, but to an increase in hip mobility and quality of life as well as sexual activity [9, 10].

Few studies have reviewed sexual function before and after THA [3, 7, 11, 12]. These studies have used questionnaires and 3D model simulations to determine the relative risk of the prosthesis, impingement, return to sexual activity and safety of sexual positions. Some of them reported cases of dislocation during sexual activity [3, 11], but details such as the position of the hip during dislocation were not determined. Therefore, the risk of impingement and instability during sexual activities has not been particularly researched. In a survey of members of the American Association of Hip and Knee Surgeons (AAHKS) [3], recommendations about sexual positions were suggested, but were based purely on the surgeon’s personal judgement. Therefore, there is currently no objective data to clearly identify which sexual positions patients perceive as satisfactory despite these recommendations. However, a motion capture study [3] described a series of sexual positions in which there is no risk of a prosthesis dislocation or impingement.

Erectile dysfunction (ED) is also a problem that affects an extensive proportion of the adult male population. For this reason, a simplified questionnaire version of the International Index of Erectile Function (IIEF) was developed. The Sexual Health Inventory for Men (SHIM) was then validated as a patient-reported diagnostic tool [13]. The SHIM is destined to assess the sexual function over the past 6 months with a score from 1 to 25 points in which a score of 21 or less may evidence an ED.

The aim of this study was to report the demographic factors, patient-reported outcome measurements (PROMs), sexual activity before and after THA and concerns related to sexual activity after THA using a short stem in a sexually active male group. We hypothesized that most patients treated by THA would be able to improve the quality of their sexual lives.

## Materials and methods

This single center study evaluated the results of male patients between 18 and 65 years of age following primary cementless short femoral stem THA (Nanos, Smith and Nephew, Marl, Germany), on a ceramic-on-polyethylene (CoP) bearing using a direct anterior approach (DAA), performed in a multi surgeon series (4 surgeons; case load > 200 THAs per year) between January 2010 and December 2019 at our institution, by using our in-clinic prosthesis registry in which patients were filtered according to inclusion criteria.

Exclusion criteria was an age younger than 18 or older than 65 years of age, female gender, the use of another short femoral stem, a follow-up less than 6 months, a hybrid or cemented THA and revision surgery.

The surgical indication for this short stem THA was osteoarthritis of the hip which failed to respond at least for 6 months to conservative treatment, patients age < 65 years, special request of the patient and /or preference of the surgeon. Patients were informed of the missing long-term data on this short stem THA and were then able to opt for a standard THA. A web-based questionnaire (via SurveyMonkey®, San Mateo, CA) was chosen for the survey to deal gently with the intimate topic of sexual activity and to allow potentially inhibited patients to participate. A total of 323 patients could be identified, whereas 227 patients were excluded because electronic contact was not possible due to German data protection regulations, leaving a total of 96 patients available for the web-based query.

**Fig. 1:**
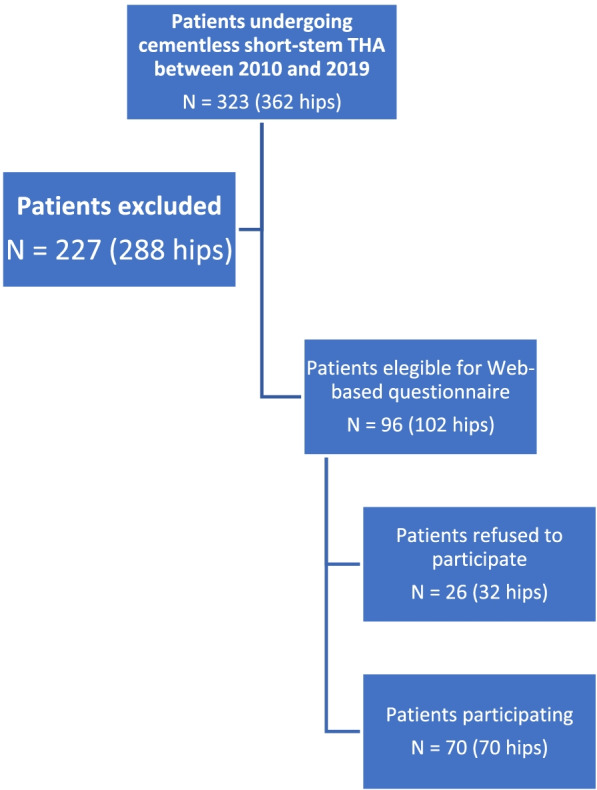
Flowchart illustrating Patient enrollment

Twenty-six patients refused to participate in the survey, resulting in a total of 70 patients (70 hips) who completed in the final web-based survey (Fig. 1).

The questionnaire used for the survey consisted of 5 parts: demographic factors, patient-reported outcome measurements (PROMs), sexual activity before and after THA and concerns related to sexual activity after THA, and the SHIM questionnaire in German version.

Demographic data included sex, age, time period after THA, diagnosis and Body Mass Index (BMI). Patient reported outcomes were assessed preoperatively as a matter of routine using the modified Harris Hip Score (mHHS) [14] and visual analogue scale (VAS) for pain. Both were then reevaluated in addition to the VAS for satisfaction at the latest follow-up.

The part of the questionnaire related to sexual activity before and after THA included the questions: does the hip pathology prior to THA affect sexual activity? (and if so, what was the most important reason); the frequencies of sexual activity preoperatively and at the latest follow-up; the time of resumption of sexual activity after THA; difficulty with leg positioning during sexual activity after THA; changes in sexual position after THA and the most common coital position before and after THA. The twelve most common coital positions and their illustration were adapted from a survey of members of the American Association of Hip and Knee Surgeons [3].

Questions on concerns related to sexual activity after THA addressed: the biggest problem during sexual activity; stress level related to sexual activity; importance of increasing sexual activity after THA, source of information on sexual activity; reasons for not communicating with a doctor on sexual activity; and the question of greatest interest. The evaluation of the questions if increasing sexual activity was rather unimportant in the decision to undergo THA was scored with 0 = completely unimportant and 10 = very important.

The ethics commission of the Landesaerztekammer Baden-Wuerttemberg, Germany approved all procedures (F-2019- 006), and the study was conducted in accordance with the Helsinki Declaration of 1975, as revised in 2008.

## Statistics

Means and standard deviations were reported for continuous variables. Differences between group means were calculated using the ANOVA test with the Bonferroni and Tukey correction. Differences between pre- and postoperative data were examined with a paired *t*-test and Wilcoxon signed-rank test. To evaluate rankings, an average ranking was calculated for each response option. We considered *p-*values of < 0.05 to be statistically significant. Statistical analysis were conducted using SPSS Statistics® (IBM SPSS Statistics for Windows, version 26.0.0; IBM Corp. Armonk, NY, USA).

## Results

### Descriptive results

A total of 70 patients were enrolled in the study. The mean age was 52.98 ± 4.89 (32–60) years, and the mean body mass index (BMI) was 27.86 ± 3.19 (20–36) kg/m^2^ (Table 1). The mean follow-up was 50 ± 31 (6–143) months. Two patients required a surgical revision (one due to persistent secretion and one due to aseptic implant loosening).

**Table 1:** Patient demographic data. SD: Standard Deviation

Characteristics	Value
Total no. of patients	70
Total no. of hips	70
Age (mean, SD)	52.98 ± 4.89
BMI (mean, SD)	27.86 ± 3.19
Laterality, n (%)	
right	46 (66%)
left	24 (34%)
Follow-up in months (mean, SD)	50 ± 31
Diagnosis, n (%)	
Osteoarthritis	43 (62.8%)
Dysplastic hip	21 (29.8%)
Rheumatoid arthritis	2 (2.2%)
Osteonecrosis of femoral head	4 (5.2%)
Revision, n (%)	2 (4%)
Persistent secretion	1 (2%)
Aseptic Implantat loosening	1 (2%)

#### Analysis of pre- versus postoperative patient-reported outcome score measurements

Postoperatively, a significant improvement in PROMs was observed. The mHHS improved significantly from 30.8 ± 14.4 (5.5–59.4) preoperatively to 96.6 ± 7.2 (35.2–100) points at last follow-up (*p* < 0.0001). There was a statistically significant improvement in pain VAS from 8.2 ± 1.4 preoperatively to 0.6 ± 1 at the last follow-up (p < 0.0001). Satisfaction of patients who underwent surgery was 9.22 ± 2.07 (1–10), with 95.7% (*n* = 70) of patients who would undergo surgery again.

#### Analysis of sexual activity before THA

**Fig. 2:**
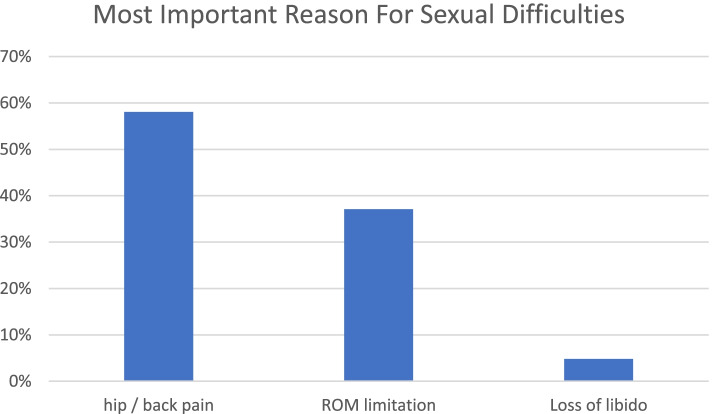
Main cause of difficulty with sexual activity after onset of symptoms. ROM, range of motion

Analysis of sexual activity data before and after THA revealed that 52% (*n* = 49) of respondents had difficulty with sexual activity after onset of symptoms. Half (*n* = 35) reported discomfort. The main cause was pain in the back or hip area (58.5%, *n* = 41) rather than limitation of the range of motion (ROM; 37.1%, *n* = 26). It was also reported a loss of libido as a minor relevant cause (2.1%, n = 3) (Fig. 2.).

#### Sexual activity after THA

**Fig. 3:**
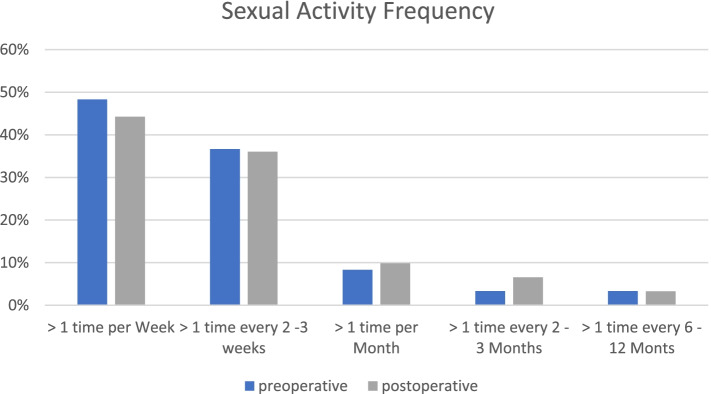
Frequency of sexual activity before and after THA

The average duration until resumption of sexual activity was 8.9 ± 9.9 (1–64) weeks. A resumption of sexual activity began after 6.3 ± 3.1 (1–15) weeks after implantation. At the last follow-up, 13 of 70 (19.1%) patients reported difficulty in positioning their operated leg during sex. There was no significant difference by the answer to the question about the change in coital position after THA (*p* = 0.17), just 10% (*n* = 7) report a change in coital positioning (Fig. 3).

#### Sexual positions

**Fig. 4:**
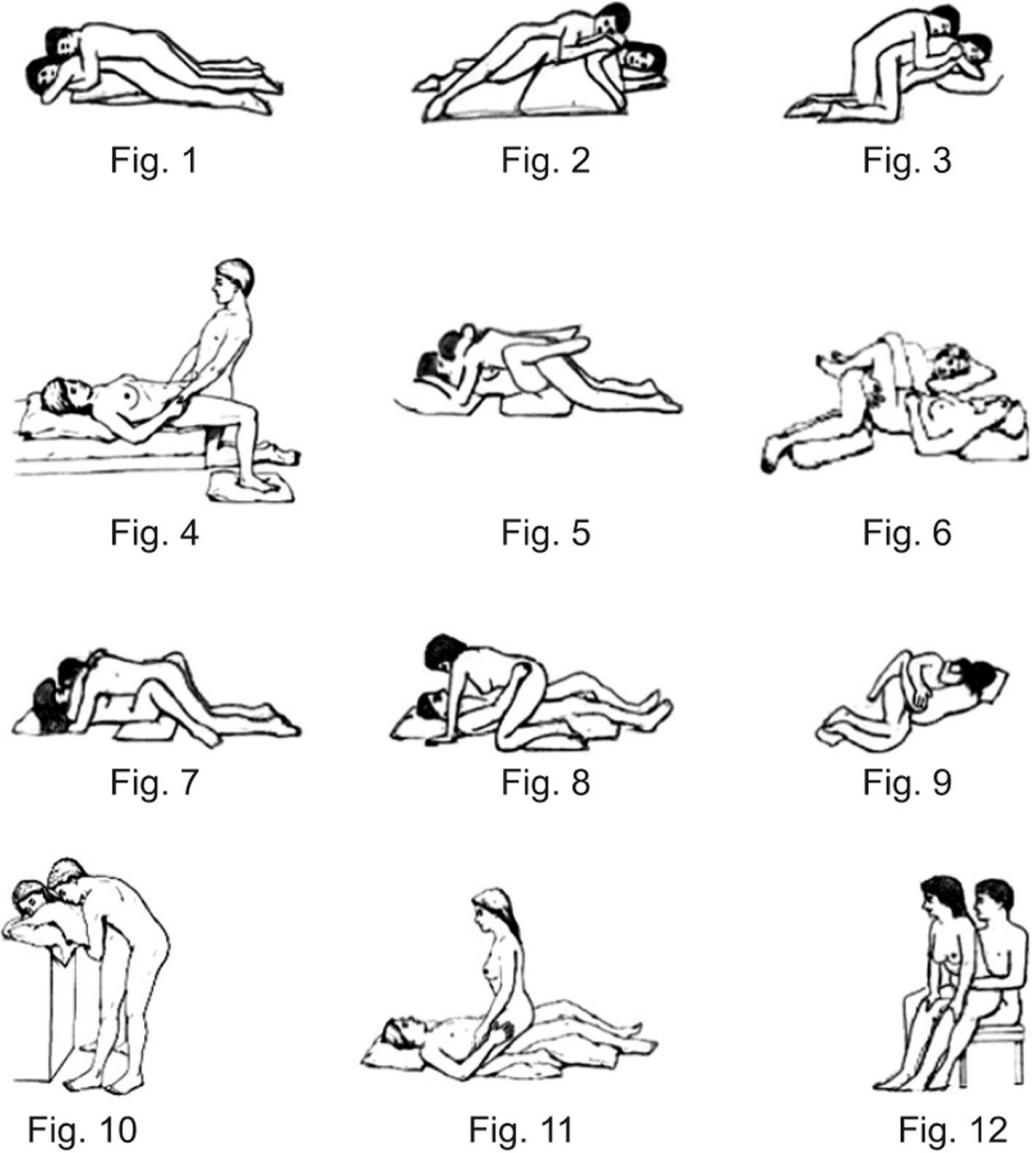
The twelve common sexual positions adapted from Dahm et al. [3]

**Fig. 5:**
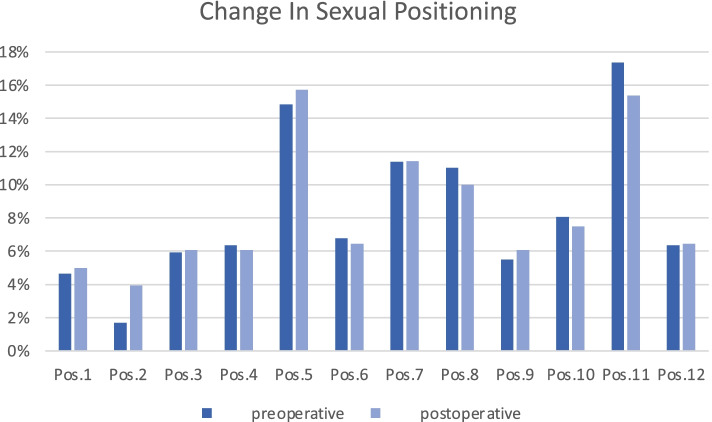
Changes between the sexual positions before and after THA

The evaluation of the twelve most common sexual positions revealed significant differences between the individual positions (*p* < 0.001). Positions 5 and 11 were the most popular. However, the changes between the positions before and after THA were not significant (*p* = 0.945) (Figs. 4 and 5).

#### Sexual satisfaction and surgery decision making

In terms of satisfaction with sex life compared to before surgery, 54.3% (*n* = 38) reported no difference. 42.9% (*n* = 30) reported an increase in satisfaction and 2.9% (*n* = 2) reported a decrease in satisfaction.

**Fig. 6:**
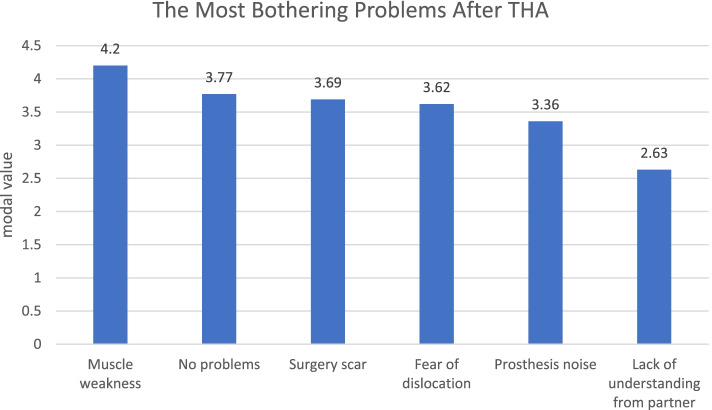
The most bothering problems after implantation of a short femoral stem prosthesis

The evaluation of the questions related to sexual activity revealed that increasing sexual activity was rather unimportant in the decision to undergo THA with 2.73 ± 2.65 (0–10). To answer the question about the most bothering problem after implantation of a short femoral stem, respondents were asked to rank possible problems. Muscle weakness was seen as the most troublesome problem, followed by the indication of no problems, problems with the surgical scar and fear of dislocation. Noise from the prosthesis tended to be less irritating for respondents, as well as a lack of understanding on the part of the partner (Fig. 6.).

When asked about the associated stress related to sexual activity and the THA, 95.7% (*n* = 67) of respondents reported any or minimal stress. One patient (1%) reported moderate stress and two patients (2.1%) reported significant stress.

Analysis of the data revealed that about one in four (26.6%, *n* = 25) adequately discussed sexual issues with their physician. One of the reasons why respondents did not talk about these issues with their physician was mainly that they did not consider it important (60%, *n* = 42), followed by the topic was too personal for them (16%, *n* = 11) and other unspecified reasons (20%, *n* = 14). 4% (*n* = 3) of the patients stated that the physician was too busy. Respondents did not state that they had asked the physician and, as a result, had not been provided with sufficient information.

When asked where patients obtained their sexual activity information after THA, 21.4% of patients (*n* = 15) researched on the Internet, 12 respondents (17.1%) got their information from medical staff and 2.9% (*n* = 2) asked other patients with the same symptoms about it; 41 patients (58.6%) cited other unspecified sources. None of the respondents obtained information on the topic from radio or television. The analysis of the question about whom patients would most likely ask for further information regarding sexual activity showed that primary (36.2%, *n* = 25) the orthopedic surgeon on charge would be the contact person. 28.7% (*n* = 20) stated that the topic was unimportant for them. The next contact person would be the family doctor (17%, *n* = 12) followed by the physiotherapist (11, 7%, *n* = 8). None of the respondents would turn to the nurse and 6.3% (*n* = 5) said they would ask other authorities. The greatest interest regarding sexual activity after THA was in relationship to safe positions, particularly in relation to dislocation (34%; *n* = 24). Furthermore, 23.4% (*n* = 16) of respondents wanted to know the right time to resume sexual activity. 10.6% (*n* = 7) of patients wanted information about durability and abrasion of the components.

98% (*N* = 69) of the patients reported a SHIM score above 21 points. One patient reported a SHIM score from 17 points.

## Discussion

Our key results are that after THA patients experienced less pain and an improvement in hip range of motion. The patients resumed their sexual activities after 6 weeks, and 89% of patients expressed a desire for more detailed and specific information on the subject. Likewise, the two main causes of difficulty in sexual activity before surgery were pain and limitation of the range of motion on the hip joint. After 4 years follow-up our patients presented a significant improvement of the mHHS from 34.1 preoperative to 92.6 after THA.

We conducted a retrospective study for which we developed and used a web-based questionnaire (via SurveyMonkey®). The reason for this decision is that in our society talking about sex is still a sensitive and embarrassing topic, so a direct interview with the patient may be less methodologically biased, but it is more difficult for patients to answer these questions personally and directly [6, 15].

The topic of sexual problems in the quality of life, in relation to stress or unhappiness in the relationship, is more easily and frankly mentioned when alone or not in the presence of another person. However, in this type of questionnaire, there is a risk that the patient may interpret the questions differently, so special emphasis should be placed on keeping the questions simple and with closed answers. Our response quote was 78.3%, which is,, one of the highest (30–80%) [6, 7 ,11, 15].

Historically, THA has been associated with improvement of the patient-reported outcome measurements (PROMs), and the development of surgical techniques and implants has led to an increase in patient expectations and postoperative improvement, with substantial attention given to biodynamic joint reconstruction for improved hip range of motion, resulting also in increased physical activity after surgery.

Concerning PROMs, the mean postoperative mHHS after one year in the general population was 88.6 (preoperative 50.8) points [16]. Patients who underwent THA under 40 years of age after 4 years follow-up showed a significant improvement of the mHHS from 34.1 preoperative to 92.6 after THA [17]. Patients with a mean age of 31 years of age showed a mean postoperative Harris Hip Score (HHS) of 84.6 points at 7 year follow-up [18]. Our data was in line with these results, even when our patient cohort was slightly older [16, 19, 20].

52% of the included patients reported some grade of difficulty with sexual activity after onset of symptoms which was slightly lower than reported rates in the literature [21]. In our study the two main causes of difficulty were referenced primarily to pain and limitation of the range of motion on the hip joint, while other studies also report fatigue and negative body image [2, 3, 6, 11, 15].

On the basis of the SHIM questionnaire, none of our patients suffered of ED.

50% of patients reported difficulties during sex. What is remarkable here, the main cause of difficulty was back and/or hip pain. Additionally, patients resumed their sexual activities after 6 weeks. This all can be presumed because of the need of less joint mobility during sex (less abduction and external rotation, which are initially limited). In addition, patients who underwent surgery with a direct anterior approach, present less risk of early postoperative dislocation, due to the preservation of the periarticular stabilizing structures [22].

In many studies THA showed beneficial effects in the increase of the frequency of sexual activity after surgery [7, 11, 23–26]. Our study does not show a significant increase in the frequency of sexual activity, as well as in the coital position before and after surgery, presumably due to the fact that the patients, because of the same discomfort before surgery, became accustomed to other coital positions that they maintained after THA.

One study reported a decrease in sexual desire before surgery, presumably because of an antalgic posture during sex, which in an attempt to modify positions may have a severe effect in the spinal column resulting in fatigue and decrease in sexual desire. Our population didn’t report loss of libido as a relevant cause [7].

For positions in which the patient raises the hip ROM, it could be shown that the risk of dislocation and impingement increases [3]. Another study could demonstrate that after surgery patients implicitly preferred positions with decreased hip ROM (positions with abduction and external rotation in a supine position) [12]. However, patients used the same positions before and after THA [3]. Our study confirmed these findings. Men’s sexual positions required less mobility and could therefore be considered safer.

An additional important aspect between THA and sexual activity is the possibility of relevant information for patients, before and after surgery. In 2014 a study found that 48% of patients preferred written instructions [27]. Other studies reported that most of the patients who underwent THA would profit from a detailed discussion with the orthopedic surgeon in charge [11, 15]. In 2004, a consensus of members of the AAHKS found that specific information was only available when the patients request for it, with a general recommendation to resume sexual activity after 1 to 3 months after THA [3]. In our cohort the majority of patients obtained the information from internet, followed from medical staff and other patients with the same symptoms. Likewise our patients consider primarily for these aspects their surgeon or family doctor, and secondarily their physiotherapist, which is in concordance with other study [3], in which 89% of patients expressed a desire for more detailed and specific information on the subject. Apart from that, the few surgeons who actually discussed the issue spent an average of less than 5 minutes on the subject [3]. We found that patients foremost concern about muscle weakness, surgical scar, fear of dislocation and noise from the prosthesis. Whereas a lack of understanding on the part of the partner was seen as unproblematic. A study in Korean population showed that the greatest sorrow of patients was a dislocation during sexual activity [2].

## Limitations

First of all, it should be mentioned that this is a web-based questionnaire survey (via SurveyMonkey), where the patient can interpret the questions differently, and that there is only the option of closed answers. Second, due to German data protection regulations from about 300 patients only data of 70 patients were collected. Third, we included only patients who underwent uncemented short femoral stem (Nanos, Smith and Nephew) THA, that reduces our cohort considerably. Fourth talking about sex in our society remains a sensitive and embarrassing topic which, despite a web-based questionnaire, may lead to information bias.

## Conclusion

THA improves quality in sexual life, in relation to less pain and improvement in the range of motion, but not in the frequency of sexual activity. The sexual positions in which increased hip flexion with abduction are combined are recommended in patients with a DAA. Despite that, no prosthesis dislocation during sex was found in our cohort. Patients do not dare to address the topic, that’s why a detailed information should be available for the patients.

## Data Availability

The datasets used and/or analyzed during the current study are available from the corresponding author on reasonable request.

## Abbreviations

AAHKS: American Association of Hip and Knee Surgeons
ANOVA: Analysis of Variance
BMI: Body Mass Index
DAA: Direct Anterior Approach
ED: Erectile dysfunction
IIEF: International Index of Erectile Function
mHHS: modified Harris Hip Score
PROMs: Patient-Reported Outcome Measurements
ROM: Range of Motion
SHIM: The Sexual Health Inventory for Men
THA: Total Hip Arthroplasty
VAS: Visual Analog Scale
WHO: World Health Organization
